# Maximizing friction by liquid flow clogging in confinement

**DOI:** 10.1140/epje/s10189-022-00208-z

**Published:** 2022-07-11

**Authors:** Shan Chen, Zhenjiang Guo, Hongguang Zhang, Ignacio Pagonabarraga, Xianren Zhang

**Affiliations:** 1 State Key Laboratory of Organic-Inorganic Composites, Beijing University of Chemical Technology, Beijing, 100029 China; 2 Institute of Automation, Chinese Academy of Sciences, Beijing, 100190 China; 3 CECAM Centre Européen de Calcul Atomique et Moléculaire, Ecole Polytechnique Fédérale de Lausanne (EPFL), Batochimie, Avenue Forel 2, 1015 Lausanne, Switzerland; 4 Department of Condensed Matter Physics, Faculty of Physics, University of Barcelona, C. Martí I Franquès 1, 08028 Barcelona, Spain; 5 UBICS University of Barcelona Institute of Complex Systems, Martί i Franquès 1, E08028 Barcelona, Spain

## Abstract

In the nanoscale regime, flow behaviors for liquids show qualitative deviations from bulk expectations. In this work, we reveal by molecular dynamics simulations that plug flow down to nanoscale induces molecular friction that leads to a new flow structure due to the molecular clogging of the encaged liquid. This plug-like nanoscale liquid flow shows several features differ from the macroscopic plug flow and Poiseuille flow: It leads to enhanced liquid/solid friction, producing a friction of several order of magnitude larger than that of Couette flow; the friction enhancement is sensitively dependent of the liquid column length and the wettability of the solid substrates; it leads to the local compaction of liquid molecules that may induce solidification phenomenon for a long liquid column.

**Graphical abstract:**

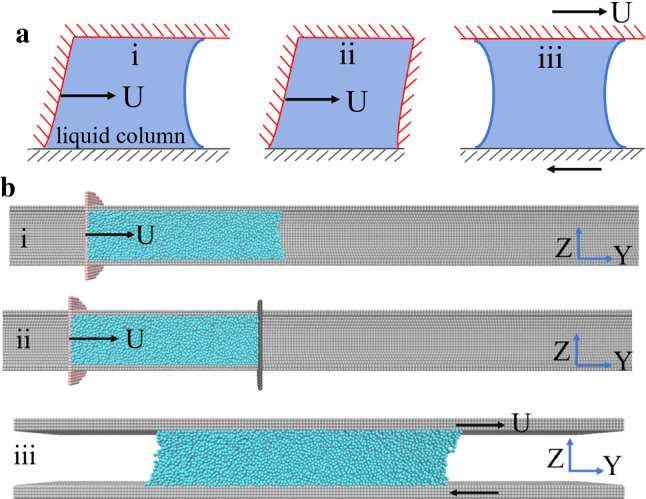

## Introduction

The dynamics of nanoconfined liquids are essential to various processes such as lubrication, filtration, energy storage and other biological scenarios [1–10]. In the nanoscale regime, transport properties show strong qualitative deviations from their bulk counterparts [11, 12]. When reducing the system size to the nanoscale, fundamental questions regarding the liquid–solid friction and shear properties remain unclear. Even for one of the simplest systems, i.e., a nonpolar molecular liquid, confined between two atomically smooth surfaces of a distances below 10 nm, reported measurements of friction span from superlubricity to moderate or high friction [13–18].

The liquid/solid frictions become much more complicated by the occurrence of solid–solid contacts. Many practical systems, such as wear, micropitting, and scuffing, operate under the particular region of liquid lubrication called mixed lubrication [19]. The lubrication regime between boundary lubrication and hydrodynamic lubrication is termed as partial lubrication or mixed lubrication. The key feature of mixed lubrication is that both hydrodynamic lubrication and asperity contacts have to be present simultaneously. At present, we have only a very limited understanding of the mixed lubrication region [20]. Compared to hydrodynamic lubrication, predicting mixed lubrication is rather difficult, because it includes the complex interaction among solid/solid contact, space confinement and fluid/solid friction. Some important questions in understanding mixed lubrication remain unsolved [19], e. g., what is the flow behavior for fluid entrained between colliding asperities, in particular for the confined film having a thickness of molecular dimension?

**Fig. 1:**
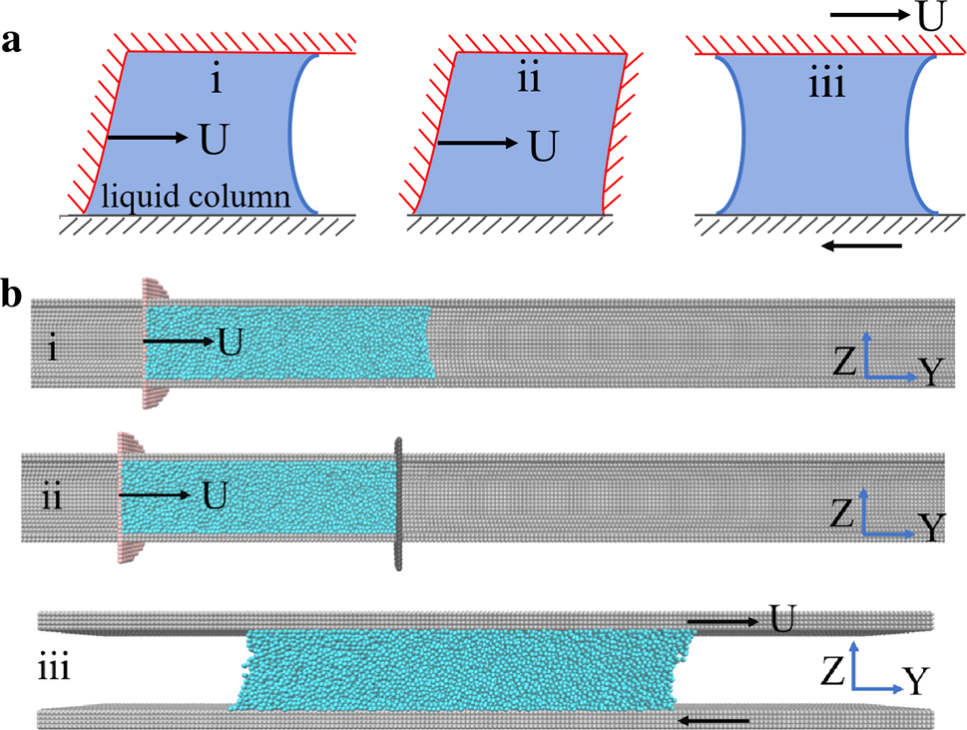
Our model for mixed lubrication with several flow types (**a**) and typical snapshots for our MD simulations on these flow types (**b**). Three different flow types were compared in this work: (i) plug-like nanoscale liquid flow with a single driving piston. The piston was placed on the left of the liquid column and exerted a driving force that causes the motion of the liquid column. (ii) Plug-like flow with two pistons. Two pistons were placed on both ends of the liquid column. The driving piston was added on the left to move the liquid column to provide the driving force, while the freely movable piston (in black) was added on the right to eliminate the capillary force. (iii) Couette flow, in which a simple steady shear flow is produced when two parallel plates move at opposite velocities

Here we used molecular dynamics (MD) simulations to address this issue through simulating flows of Lennard–Jones (LJ) chain-like liquid encaged in a solid cylindrical nanopore with atomically smooth surfaces. To simulate the influence of nearby solid/solid contacts on the liquid/solid friction, we propose a simplified mode for the contacts among surface asperities, described as a movable driving piston (Fig. 1). In this simplified geometry, the piston drives the plug-like flow of the confined liquid column, and the driving force is balanced by the liquid/solid friction. To separate the solid/solid friction from that of liquid/solid friction, we omit the former in this work by setting the interaction between the solid piston and the pore wall to zero. Our simulations reveal the existence of an additional variable, i.e., molecular clogging, that essentially affects liquid/solid friction. It is the solid–solid contact that induces molecular clogging for strongly confined liquid, leading to a new flow structure in the nanoscale. We subsequently demonstrate that this type of plug-like nanoflows shows several features that are entirely different from macroscopic plug flow and Poiseuille flow.

## Model and simulation details

In this work, we use Molecular Massively Parallel Simulator (LAMMPS) [21] to investigate how the molecular clogging and spatial confinement cooperatively affect the friction force between a liquid column and confining solid substrates. To mimic the liquid/solid friction under molecular clogging, the motion of the liquid column confined in a small cylindrical channel is generated by a driving solid piston, which moves at a prescribed velocity (Fig. 1b).

In our simulations, the non-bonded interaction, ${U}_{ij}\left(r\right),$ between any two atoms of types *i* and *j* is represented by the 12–6 Lennard–Jones (LJ) potential ${U}_{ij}\left(r\right)=4{\varepsilon }_{ij}(({\frac{{\sigma }_{ij}}{r})}^{12}-({\frac{{\sigma }_{ij}}{r})}^{6})$, where r is the distance between the beads, ${\sigma }_{ij}$ is the size parameter, ${\varepsilon }_{ij}$ is the energy parameter. Here, *i* and *j* can be wall (*w*), piston (*p*), and liquid (*l*) atoms. All variables, unless specified, will be presented in reduced units, with length and energy scales denoted as $\sigma$ = $\sigma_{ll}$ and $\varepsilon = \varepsilon_{ll}$. For the LJ potential, the cutoff radius ${r}_{c}$ was set to 2.5σ. The liquid is modeled by chain-like LJ molecules, each containing eight LJ atoms. The intramolecular bonded interactions of the chain-like molecules are modelled by a finite extensible nonlinear elastic (FENE) potential [22]$E=-0.5K{R}_{0}^{2}Ln(1-({\frac{r}{{R}_{0}})}^{2})$. The bond length $r$ has a maximal length of ${R}_{0}=$ 1.5*σ*, and the spring constant $K=30 \varepsilon /{\sigma }^{2}$. Note that in our simulation runs we used LJ units, and in order to convert to the real units, the LJ particles can be considered as argon atoms, e.g., *σ* = 3.41 Å and *ε* = 10.30 meV [23].

To mimic the liquid/solid friction in mixed lubrication, we built a typical system as sketched in Fig. 1a, in which we considered three distinct geometries: (i) The confined liquid column is pushed by a driving solid piston at a prescribed velocity; (ii) the same as in (i) except introducing two pistons, with the left side one exerting a prescribed pushing velocity and the other being freely movable to cancel the contribution of capillary forces to the liquid column motion; (iii) the Couette flow of a thin liquid slab that is driven by two flat confining solid walls moving in opposite directions. Comparing different types of flows, we are able to illustrate how the resulting motion and involved friction forces are affected by both geometrical and the exerted driving velocity. To separate the solid–liquid friction from the solid–solid friction, the interaction between pistons and capillary channel (in cases i and ii) was set to zero. Figure 1b depicts the typical snapshots for the flows corresponding to the geometries involved in our simulations. In Fig. 1b, cases i and ii display typical configurations leading to a plug-like nanoflow within a strongly cylindrical solid channel, and case iii shows the shape of the liquid slab subject to a Couette flow. The size of the three-dimensional simulation box was set to 40 *σ* × 460 *σ* × 40 *σ* (cases i and ii in Fig. 1b) and 15 *σ* × 200 *σ* × 40 *σ* (case iii in Fig. 1b), with periodic boundary conditions applied to all directions. In detail, the Couette flow for thin liquid slab of 2016 liquid molecules (Fig. 1b) was generated by moving simultaneously the upper and lower walls at a same velocity *U* but along the opposite directions. Each smooth wall contained 11,940 atoms that form layers, and the minimal distance between the two walls was set to 18 *σ*. In cases of plug-like nanoflow, the radius of each piston that contained 793 solid atoms was set to 16 *σ*, somewhat larger than the pore size ranging from 9 to 11 *σ*, to prevent the liquid column from flowing out. Unless otherwise stated, 1992 liquid molecules that formed a liquid column were placed inside the capillary tube with a length of ~ 64 *σ* and a pore size of 9 *σ*.

The wettability of capillary tubes and flat walls can be either hydrophilic (by setting the interaction parameter between liquid and solid atoms ${\varepsilon }_{lw}\hspace{0.17em}$= 0.8) or hydrophobic (${\varepsilon }_{lw}$=0.4), while that of pistons was set to almost neutral (${\varepsilon }_{lp}$ = 0.6). In our simulation, the solid components (including pistons and the pore walls) were set to rigid [24]. This treatment along with liquid thermostat removes the heating effect generated by friction and liquid viscosity and thus simplifies our analysis. NVT was used for the liquid slab (case iii in Fig. 1b) and liquid column (cases i and ii in Fig. 1b) to update atom positions and velocities at each time step. In our simulation runs, typically 5.2 ns was required to reach the steady state, followed by a production time interval up to 32 ns to obtain liquid velocity profiles. The velocity Verlet algorithm with a time step of 0.003 $\tau$ was used in MD simulations for the integration of equations of motion, and a Nosé–Hoover thermostat with a time constant of 0.3 $\tau$ was used to maintain the liquid temperature to *k*_*B*_*T* = 0.8 *ε*, where *k*_*B*_ is the Boltzmann constant. Here $\tau =\sigma \sqrt{m/\varepsilon }$ and $m$ is the mass of liquid atom. At the thermodynamic conditions, the fluid is found in a liquid state [25–27].

## Results and discussion

### Enhanced friction for plug-like nanoflow in strongly confined liquids

**Fig. 2:**
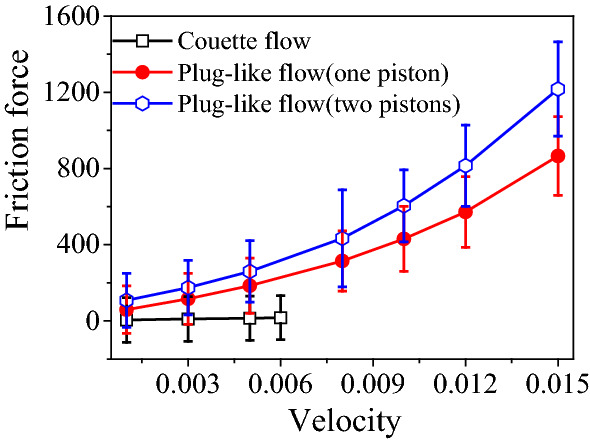
The friction force changes with the driving velocity and flow type. In this figure, the pore diameter of cylindrical channels was set to 18 *σ*, and in the case of the Couette flow the distance between two confining substrates was also 18 *σ*

First, we consider hydrophilic channels and study how the friction force between the encaged liquid column and the solid substrate changes with flow type. We compare the resulting friction for the three different flow types as well as their variation with fluid velocity (Fig. 2). In general, as depicted in Fig. 2, the friction force for the plug-like nanoflow under strong confinement is substantially larger, up to 1–2 orders of magnitude, than the one measured for Couette flow.

Since the fluid column moves under the action of the piston without inertia, the friction force exerted between the piston and the fluid, ${F}_{\mathrm{philic}}^{f}$, can be obtained from force balance, ${F}_{\mathrm{philic}}^{e}-{F}_{\mathrm{philic}}^{f}+{F}_{\mathrm{philic}}^{c}=$ 0, where ${F}_{\mathrm{philic}}^{e}$ stands for the force exerted by the driving piston on the left-hand side of the slab, which can be obtained by directly summing up all the interaction force between the piston and the liquid, and ${F}_{\mathrm{phili}}^{c}=2\pi r\gamma \mathrm{cos}{\theta }_{\mathrm{philic}}$ corresponds to the capillary force of the liquid/gas slab interface. To this end, the surface tension and equilibrium contact angle are measured through independent equilibrium simulations.

Specially, for the contact angle measurements, we conduct NVT MD simulations letting equilibrate a droplet of the corresponding liquid sitting on a flat solid substrate with the same hydrophilicity as the channel wall, and then, the contact angle was extracted from the droplet shape. For the given interaction parameters between liquid and the substrate ${\varepsilon }_{lw}$ = 0.8, 0.6 and 0.4, the measured contact angle is 52.1°, 86.85° and 115.65°. Additional MD simulation runs including a liquid slab with flat vapor/liquid interfaces in a 50 *σ* × 50 *σ* × 60 *σ* simulation box were performed to determine the surface tension [28, 29] by $\gamma =\int_{-\infty }^{+\infty }{P}^{zz}(z)-({P}^{xx}(z)+{P}^{yy}(Z)/2)dz$, extracted from the measure of the components of the pressure tensor perpendicular, ${P}^{zz}(z)$, and parallel, ${P}^{xx}(z)$ and ${P}^{yy}(z)$ to the interface. For liquid model used here, the determined surface tension is 1.02. Thus, for ${\varepsilon }_{lw}$=0.8, 0.6 and 0.4, which correspond to the contact angle of 52.1°, 86.85° and 115.65°, the calculated capillary forces are ${F}^{c}=$ 35, 3.4 and − 25 in reduced units. Therefore, based on the force balance analysis the friction force can be determined (Fig. 2).

For the flow type (ii) (case ii in Fig. 1b), two pistons were introduced: one is the driving piston on the left side of the liquid column, and the other is placed on the right that are freely movable. The given velocity was applied to the left piston to push the liquid column, while the freely moving right piston eliminates the capillary force, i.e., ${F}_{\mathrm{philic}}^{c}=0$. In this geometry, force balance reduces to ${F}_{\mathrm{philic}}^{e}-{F}_{\mathrm{philic}}^{f}=0,$ and the friction force equals the force applied to the left piston by the liquid.

For the Couette flow (case iii in Fig. 1b), the flow is induced by the shear stress from two confining solid walls moving with the same velocity *U* but in opposite directions. When the liquid column reaches its steady state, the friction force between the liquid slab and the solid walls can be directly measured.

The obtained friction forces are compared in Fig. 2. It is found that for the plug-like nanoflow (cases i and ii in Fig. 1b) under extreme confinement, the measured friction force can be two orders of magnitude larger than that of Couette flow (case iii). For example, at a piston velocity of *U* = 0.005, the friction force is 13.8 for Couette flow but 259.64 for plug-like flow with two pistons (Fig. 2). Furthermore, the two types of flows show different dependence on velocity *U*. In contrast to the Couette flow, the plug-like nanoflow clearly shows a sharp and nonlinear increase with velocity. The simulation results also show that as expected, the presence of the piston on the right increases the friction on the liquid column, partly because of the absence of capillary force (Fig. 2). Therefore, the presence of two pistons leads to a larger friction force on the liquid column than when it is in contact with a single forcing piston.

### The mechanism of enhanced friction force for the plug-like nanoflow: impact of new flow structure

**Fig. 3:**
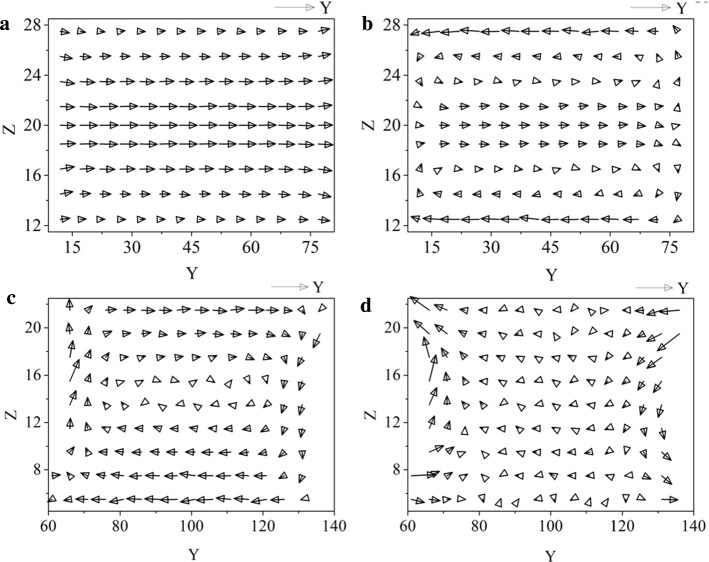
The flow structure is crucial to the generated friction force: (**a**, **b**) for the plug-like nanoflow and (**c**, **d**) for the Couette flow. **a** Velocity distribution for the plug-like nanoflow with two pistons. **b** Distribution of relative velocity for the liquid flow with respect to the driving piston, namely $V\prime \left( {y,z} \right) = V\left( {y,z} \right) - U$. In this figure, the piston velocity *U* = 0.01. **c** Velocity distribution for the Couette flow. **d** Distribution of relative velocity for the Couette flow. In Figure (**c**, **d**), the velocity *U* applied on the upper and lower solid substrate is 0.005 and − 0.005

In order to gain insight for the mechanism leading to the enhanced friction force for the plug-like nanoflow, we analyze the velocity profile of the liquid column. Figure 3a displays averaged velocity profile along *Y* axis and *Z* axis (${V}_{y}$ and ${V}_{z}$) in each individual cell of $2 \sigma \times 5 \sigma$ was determined by summing up over 10^5^ configurations (Fig. 3a). Figure 3b shows the relative velocity of the fluid with respect to the driving piston, which displaces at constant speed *U*, namely $V^{\prime}\left( {y,z} \right) = V\left( {y,z} \right) - U$. To be clear, velocity profile of the plug-like nanoflow is similar to the Poiseuille flow, showing a shape of parabolic curve, but the difference between them is obvious: the molecular clogging induces a forced relative motion between the wall and the fluid layer closest to it, which leads to a large molecular friction. Moreover, the profile of relative velocity shows an unexpected tendency: the largest relative motion appears near the whole channel wall–liquid interface although there exists the strong interaction between the liquid and the static capillary walls. The net forward flow in the center of channel cancels the backward motion of the liquid close to the solid walls (Fig. 3b). Moreover, the pushing piston and the liquid/vapor interfaces induce the development of two vortical fluid rolls in the upper and lower part of the channel. Such fluid structure, where convection is strongly hindered because of the strong confinement, is distinct from the usual macroscopic Poiseuille flow in long, open-ended channels.

In particular, the piston encages the nanoflow, suppressing the relative motion of liquid molecules far from the interface and thus inhibiting shear flow (Fig. 3b). This effect is called clogging here. In this study, the clogging induced by the presence of the piston suppresses internal relative motion inside the liquid and inhibits the shear flow.

For the Couette flow, the profile of liquid velocity $V\left(y,z\right)$ and that of relative velocity $V^{\prime}\left( {y,z} \right)$ are given in Fig. 3c and d, respectively. The profile of relative velocity is determined by $V^{\prime}\left( {y,z} \right) = V\left( {y,z} \right) - v_{y}^{T}$ with theoretical velocity profile ${v}_{y}^{T}$ along the Z axis ${v}_{y}^{T}=U(z-\frac{H}{2})/\frac{H}{2}$. Here *H* is the inter-substrate distance. As shown in Fig. 3d, the profile of relative velocity indicates that close to solid walls, relative velocity of the fluid is nearly 0, indicating the most liquid molecules near the substrates have the nearly same velocity as the moving substrates. Therefore, liquid molecules in Couette flow show a caterpillar-like motion (Fig. 3c) [30]. Figure 3d also shows that large relative velocity only appears in the vicinity of the contact line and on the vapor–liquid interface. Thus, the relative motion only exists in the contact line region, demonstrating that the friction force mainly comes from the contact line. Although the friction of Couette flow can come from both the liquid viscosity and contact line, in our study the contact line friction dominates due to the molecule model used and the short length of the liquid column.

The comparison of flow morphology between plug-like nanoflow (Fig. 3b) and Couette flow (Fig. 3d) indicates that for the plug-like nanoflow, the sliding friction occurs over the whole liquid–solid interface at which the liquid molecules come in contact with the capillary. Thus, the friction force for the plug-like nanoflow comes from the contact area, rather than the contact line. Therefore, the friction force of plug-like nanoflow is predominantly governed by the interface contribution, since the viscous contribution from the bulk of the liquids and the contact line friction are minimized. This friction force is strongly controlled by the confinement induced by the piston, which forces the liquid column to move uniformly for the fluid in contact with the solid wall and extends into the liquid column. This characteristic is responsible for the high friction force observed in Fig. 2.

Although the strongly confined liquid in piston-forced nanoflow has a restricted relative motion and displays quantitative features analogous to those of solid-like displacement in the region close to the moving piston, no liquid–solid transition is required for generating the observed high friction. Rather, it is due to the relative motion inhibition force by the confining piston, which suppresses relative internal motion of liquid, and which we refer to as clogging.

The difference of the piston-induced flow structure studied here with the macroscopic plug flow, even if we refer to it the plug-like nanoflow, is worth being highlighted. First, the nanoflow induces an unexpected high friction. Second, for the ideal plug flow, the liquid in a given cross-section has identical velocity and backmixing is missing. However, plug-like nanoflow shows a different flow structure (Fig. 3b): the velocity profile becomes heterogeneous and two vortical fluid rolls appear for forced fluid mixing.

The plug-like nanoflow is also different from the Poiseuille flow. For Poiseuille flow, the fluid layer closest to the wall adheres to it, and the relative velocity must vanish due to the no-slip boundary condition. For the piston-induced flow patterns, however, a different picture emerges due to molecular clogging: the molecular clogging induces a forced slip between the wall and the closest fluid layer, which leads to an enhanced friction depending on the contact area. As a result, the formation vortices was to compensate for the interfacial friction (Fig. 3b).

### Clogging and confinement cooperatively induce the interface friction

In the previous section, we have concluded that the presence of the driving piston, which induces clogging, along with the strong confinement that forces a strong decrease of the velocity away from the channel center, cooperatively results in the new flow structure shown in Fig. 3 and leads to enhanced friction.

**Fig. 4:**
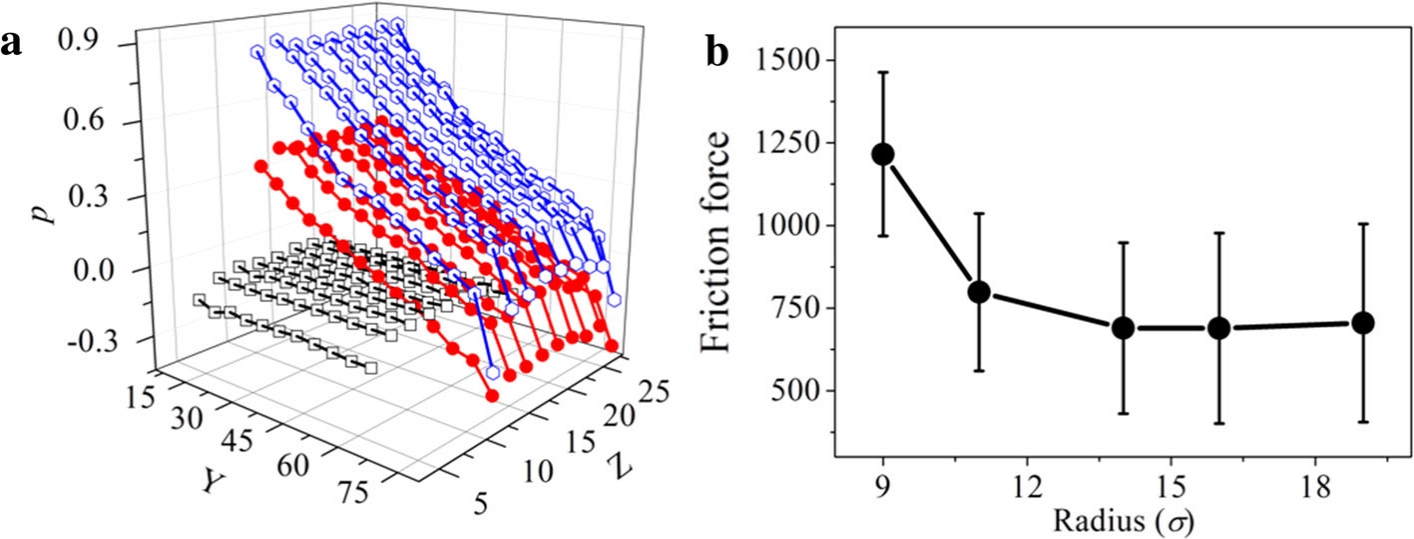
**a** Distribution of pressure, determined by ${(P}_{xx}+{P}_{yy}+{P}_{zz})/3$, along the liquid column. In this figure, the piston velocity in cases i and ii and the velocity for the upper and lower substrates in case iii were both set to 0.005. The symbol square box, filled circle and Hexagon represent Couette flow, plug-like flow (one piston) and plug-like flow (two pistons), respectively. **b** The friction force for plug-like nanoflows changes with the radius of the capillary tube. In this figure, the plug-like flow was generated by two pistons, and the velocity for the driving piston was set to 0.015

The piston inhibits the emergence of a fully developed Poiseuille flow, leading to a more homogeneous displacement of the liquid in a region which penetrates from the piston. In order to understand how the high friction is generated by such inhibited relative motion, we have compared the pressure gradient along the liquid column from plug-like nanoflow and Couette flow. We have computed the symmetric pressure tensors, namely ${P}_{xx}$,${P}_{yy}$,and ${P}_{zz}$ of the liquid column. For a fluid in motion, the three normal pressure tensors may be different from each other, and in this work we focus on its trace, namely $p\equiv ({P}_{xx}+{P}_{yy}+{P}_{zz})/3$ [31], which can be used as an indicator of the friction force distribution. As shown in Fig. 4a, the pressure drops for the plug-like nanoflow with one or two pistons which is much higher than the corresponding one for Couette flow. For the plug-like nanoflow, the sharp and nonlinear gradient of the pressure drop along the channel correlates with the measured enhanced friction and also indicates the uneven compression of the liquid along channel imposed by the pushing piston. For the Couette flow, however, the pressure is almost uniform everywhere. The comparison among various flow types indicates that in our work the difference in the frictional pressure drop does not result from fluid viscosity and channel roughness. Instead, the clogging-induced flow structure is crucial for determining the corresponding pressure drop.

Figure 4b displays the decrease in friction as the radius of a hydrophilic capillary channel increases. The liquid column is confined between two pistons, and the driving one moves at a speed 0.015. The increase in friction for radii below 15 $\sigma$ signals the regime of strong confinement and clogging, where the relative liquid motion hindrance imposed by the pistons dominates the interaction between the liquid and confining solid. For channels with a size of several nanometers, the rolling motion is severely suppressed, enhancing clogging. As the channel size increases, the clogging weakens, resulting in a smaller friction.

### Effects of the length of the liquid column

Since the friction force for the plug-like nanoflow comes from the whole contact area, we expect that the magnitude of the measured friction force will be sensitive to the liquid column length. We have analyzed the dependence of the friction force on the liquid column length for a plug-like nanoflows guided by two pistons.

**Fig. 5:**
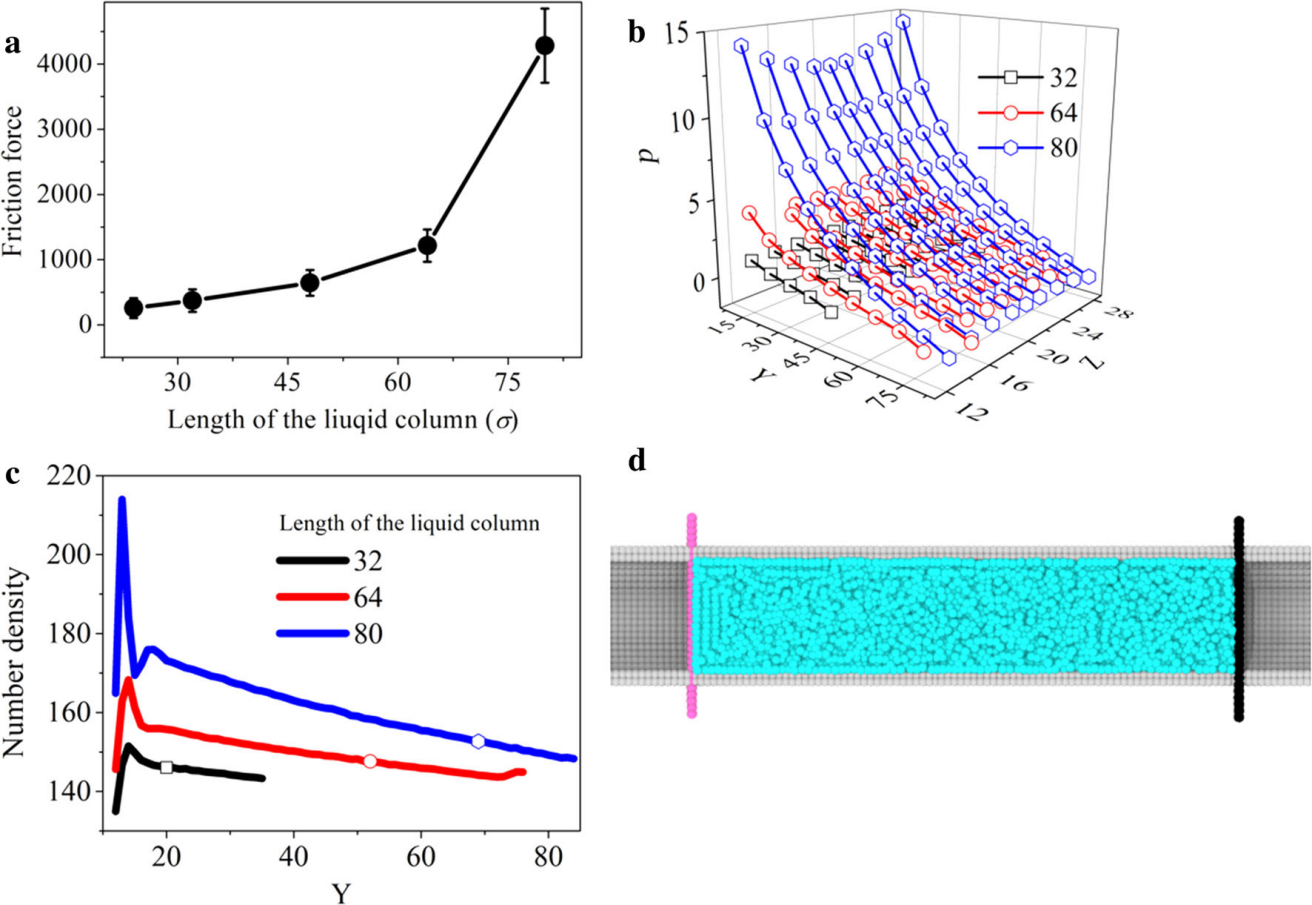
**a** The friction force changes with the length of liquid column. **b** Distribution of internal pressure along the liquid column. **c** Number density along the liquid column as a function of its length. **d** The snapshot for the length of liquid column being setting to 96*σ*

The initial length of liquid column is, respectively, set to 24 *σ*, 32 *σ*, 48 *σ*, 64 *σ* and 80 *σ*, and a constant velocity of 0.015 is applied on the left driving piston, while the right piston is allowed to freely move. Figure 5a displays the nonlinear and sharp measured friction force with the length of liquid column.

Two different aspects contribute to the nonlinear increase of the friction force. On the one hand, since the whole solid surface contributes to the friction, one expects an increase of the friction force with the liquid column length. On the other hand, Fig. 5b shows that the internal pressure of the liquid increases with the liquid column length due to the compression induced by the friction when relative motion is suppressed. The liquid compression in turn inhibits the internal rolling motion and enhances clogging, giving rise to further increase in friction force. The number density profiles, shown in Fig. 5c, indicates how such density increases with the liquid column length in response to the driving piston push.

We have also found that for a sufficiently long liquid column, liquid compaction may lead to a pressure-induced solidification for a liquid confined in a nanometric capillary. As an illustration, Fig. 5d displays a solid configuration for a 96*σ* column of length*.*

### The effect of wettability of capillary tube

**Fig. 6:**
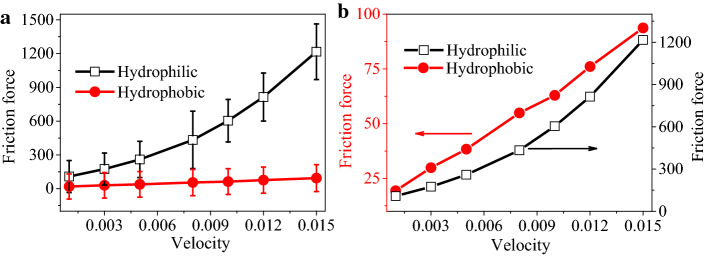
**a** The comparison of the generated friction force changes with the wetting property of capillary at the given piston velocity. The corresponding wetting property of hydrophilic and hydrophobic cylindrical capillary is 52° and 115°. **b** In order to highlight the effect of wettability of capillary, the same data are re-plotted to compare the rate of friction enhancement as a function of velocity

The interface-dominated friction mechanism predicts a substantial effect of substrate wettability on friction. Decreasing the solid wettability weakens the solvent molecule adsorption close to the capillary walls. Figure 6a quantifies and confirms this trend for a plug-like nanoflow both in hydrophilic and hydrophobic tubes (Fig. 6). The reduced attraction of the solvent molecules to the confining solid interface favors their relative motion (slip), leading to a substantial decrease in the liquid friction, which also displays a much weaker dependence on the compression induced by the piston as its velocity increases. For the hydrophilic channel, the friction-induced liquid compression leads to an increase in the rate of friction enhancement with velocity. But for the hydrophobic tube, although the friction force increases with velocity, the enhancement of friction force is weakened by the interfacial slip. The difference can be more clearly demonstrated in Fig. 6b.

## Conclusions

The forced motion of confined liquid is ubiquitous in nature. A particular example of this kind of flows takes place in the mixed lubrication. The key feature of mixed lubrication is the presence of asperity interactions at several locations while existing thin lubricant films at other locations. Although many practical systems, such as wear, micropitting, and scuffing, operate in the lubrication region, the flow behavior for liquid entrained between colliding asperities is still largely unknown. In this study, we have used molecular dynamics (MD) simulations to analyze the flow behavior of a liquid entrained between colliding asperities, in particular for the film thickness of confined fluid having molecular dimensions.

Our simulations reveal the existence of an additional mechanism, i.e., piston-induced clogging, which essentially affects and determines the liquid/solid friction. The presence of solid–solid contact induces clogging for strongly confined liquid, leading to a new flow structure in the nanoscale. This plug-like nanoflow shows several features that are qualitatively different from those observed in the macroscopic plug flow and Poiseuille flow: (i) piston-induced clogging and strong confinement cooperatively result in the flow structures with hindered relative motion that leads to enhanced friction. As the channel size increases, hence both the confinement effect and the piston-induced clogging weaken, resulting in a decrease in the friction. (ii) When piston-induced clogging is relevant, the confined liquid shows a more homogeneous, solid-like, response to the motion imposed by the piston, with a strong reduction in the relative liquid motion. Hence, the friction force comes from the whole contact area, rather than the contact line. As a result, this flow regime is characterized by a liquid/solid friction of several orders of magnitude larger than that of Couette flow. (iii) Due to clogging and high substrate friction, the friction force and internal pressure increase sharply with the length of liquid column. The liquid local compaction in turn inhibits of the liquid relative motion and enhances the liquid/solid friction. The friction-induced compaction may induce solidification for sufficiently long liquid column. (iv) The decrease in the wettability substrate reduces the adsorption of solvent molecules, leading to a substantial decrease in friction with respect to the one measured for hydrophilic substrates.
